# Exploring Factors Associated with the Psychosocial Impact of Stigma Among People with Schizophrenia or Affective Disorders

**DOI:** 10.1007/s10597-014-9800-1

**Published:** 2014-12-23

**Authors:** Piotr Świtaj, Anna Chrostek, Paweł Grygiel, Jacek Wciórka, Marta Anczewska

**Affiliations:** 1 First Department of Psychiatry, Institute of Psychiatry and Neurology, ul. Sobieskiego 9, 02-957 Warsaw, Poland; 2 Educational Research Institute, Warsaw, Poland

**Keywords:** Stigma impact, Schizophrenia, Affective disorders, Mental illness, Poland

## Abstract

Stigmatization can exert a variety of pernicious effects on the lives of persons with mental illnesses. The purpose of this study was to explore factors related to the psychosocial impact of stigma among 229 people receiving psychiatric treatment: 123 with schizophrenia [*International Classification of Diseases, 10th Revision* (ICD-10): F20] and 106 with affective disorders (ICD-10: F31–F33). In the whole sample, the factors most prominently associated with a greater impact of stigma on personal and family life were schizophrenia diagnosis, current inpatient treatment, actually experienced stigma and self-stigma. However, the patterns of predictors varied between the two diagnostic categories. For the schizophrenia group, only self-stigma significantly contributed to a stronger stigma impact. In the affective group, a more severe impact of stigma was significantly predicted by inpatient status and experienced stigma. Anti-stigma programs should address the specific features of stigmatization associated with various psychiatric diagnoses.

## Introduction

Population surveys carried out across the world reveal that despite the increase in the public’s mental health literacy, the level of social rejection of people with mental illnesses did not change for the better over the last decades, and that with respect to people with schizophrenia it actually worsened (Schomerus et al. 2012). Thus, although numerous efforts have been undertaken globally to eradicate psychiatric stigma (Sartorius and Schulze 2005), it continues to exert pernicious effects on people with mental health problems, their families, treatment providers and whole communities (Corrigan and Kleinlein 2005).

Current theoretical models conceptualize mental health stigma as a complex phenomenon involving several interrelated components, such as: stereotypes, prejudice, and discrimination (Corrigan and Kleinlein 2005), or problems of knowledge (ignorance or misinformation), problems of attitudes (prejudice), and problems of behavior (discrimination) (Thornicroft et al. 2007), or labeling, stereotyping, separation, emotional reactions, status loss, and discrimination in a situation where power is exercised (Link et al. 2004). The authors of all these models are in agreement that stigmatization cannot be fully understood without taking into account the subjective perspective of people with mental illness, who play an active and important role in this process. This subjective perspective is often referred to as personal stigma, as opposed to public stigma, i.e. the reaction of the general population towards people with mental illness. Usually, three main aspects of the personal stigma of mental illness are distinguished (Brohan et al. 2010; Gerlinger et al. 2013): (1) perceived stigma, i.e. an individual’s beliefs about the extent to which society stigmatizes the group to which he/she belongs and him/her personally as a member of a potentially stigmatized group; (2) experienced stigma, i.e. actually encountered rejection and discrimination; and (3) self-stigma or internalized stigma, i.e. the process of the internalization of stigmatizing societal attitudes, resulting in fear of discrimination, social withdrawal, feelings of shame, guilt, and hopelessness, and a decrease in self-esteem and self-efficacy. However, a recent review of measures of experiences of mental illness stigma, prejudice and discrimination found that some of them also covered elements of personal stigma which did not clearly fit into the categories of perceived, experienced and self-stigma (Brohan et al. 2010). Examples are stigma resistance, positive aspects of mental illness, impact of stigma, or stressfulness of stigma events. The authors of this review concluded that it would be useful to consider these additional constructs. In this paper, we aim to explore one of them, namely the impact of stigma as measured by a subscale of the Inventory of Stigmatizing Experiences (ISE; Stuart et al. 2008). It is defined by the developers of the ISE as “the intensity of psychosocial impact of stigma on major life domains such as quality of life, family relations, social contacts and self-esteem” (Stuart et al. 2008, p. 194). It is, then, conceived as a measure of the global effects of stigmatization on an individual’s personal and family life. While the ways in which stigma interferes with specific psychosocial outcomes have already been repeatedly demonstrated (Gerlinger et al. 2013; Livingston and Boyd 2010), identifying factors contributing to the overall psychosocial impact of stigma may constitute a valuable expansion of this body of knowledge and may further help to guide anti-stigma programs.

It seems of particular interest to determine how stigma impact depends on the type of mental illness. It is universally believed that the burden of stigma is particularly high among people diagnosed with schizophrenia (Read et al. 2006; Sartorius and Schulze 2005). However, a still growing amount of evidence indicates that it may also be very harsh for people with affective disorders (Brohan et al. 2011; Hawke et al. 2013; Lasalvia et al. 2013). A better understanding of the specific features of stigmatization associated with these two diagnostic categories would enable the elaboration of more tailor-made interventions that may prove to be more effective than those focusing on mental illness in general (Angermeyer and Matschinger 2003).

The specific objectives of the present study were as follows: a) to examine which socio-demographic and clinical variables are most prominently related to the intensity of the impact of stigma reported by people with mental illnesses; b) to determine how various aspects of personal stigma (i.e. perceived, experienced or self-stigma) contribute to the impact of stigma; and c) to investigate the differences in the magnitude and predictors of stigma impact between persons with schizophrenia and persons with affective disorders.

Although our analyses were exploratory in nature, we stated some preliminary hypotheses. First, based on a systematic review of the studies of public attitudes, which revealed that rejection towards people with schizophrenia is generally more pronounced than towards people with mood disorders (Angermeyer and Dietrich 2006), we expected a diagnosis of schizophrenia to predict a higher impact of stigma. Second, since it has been found in longitudinal studies that the harmful effects of perceived stigma on the well-being of people with mental illness are weaker that those of self-stigma (Ritsher and Phelan 2004) and are substantially reduced or become non-significant when actual rejection experiences are controlled for (Link et al. 1997; Markowitz 1998), we hypothesized that internalized and experienced stigma would display more robust relationships with stigma impact than perceived stigma. Third, we assumed that the impact of stigma would be greater among respondents with more severe psychiatric symptoms, which may increase the probability of encountering social rejection (Farina 1998) and have been demonstrated to be significantly related to personal stigma in the majority of relevant studies (Gerlinger et al. 2013; Livingston and Boyd 2010). Fourth, we reasoned that a longer duration of illness would be associated with a greater exposure to stigmatization and, as a result, with its stronger psychosocial impact. Finally, given that psychiatric hospitalization is regarded as an especially stigmatizing form of treatment (Falk 2001), we expected that participants from inpatient wards would report a more intense impact of stigma than those under outpatient, community or day care. We made no specific predictions regarding socio-demographic variables, because in recent literature reviews (Gerlinger et al. 2013; Livingston and Boyd 2010) none of them showed consistently significant associations with personal stigma.

## Methods

### Sample

Study participants were recruited from various mental health care facilities of the Institute of Psychiatry and Neurology (IPiN) in Warsaw (Poland). The inclusion criteria were as follows: (1) diagnosis of schizophrenia (F20) or affective disorders (F30-F33) according to the criteria of *the International Classification of Diseases, 10th Revision* (ICD-10); (2) age over 18 years old; (3) written, informed consent to participate in the study; and (4) a stable mental condition, according to the treating psychiatrist, enabling understanding and accurate answering of the questions in the questionnaires. Individuals with active drug or alcohol dependence, organic brain disease, severe cognitive deficits or documented mental retardation were excluded.

Out of 281 persons who were asked to participate in the study, 52 (18.5 %) refused. The final sample included 229 participants—123 (53.7 %) with schizophrenia and 106 (46.3 %) with affective disorders. In the affective group, slightly more than a half of respondents (*n* = 56, 52.8 %) were diagnosed with bipolar affective disorder (ICD-10: F31), 12 (11.3 %) had a diagnosis of depressive episode (ICD-10: F32), and 38 (35.8 %) had experienced recurrent depressive disorder (ICD-10: F33).

### Measures

Socio-demographic and clinical background characteristics of the participants (including age, sex, level of education, marital status, living situation, employment status, duration of illness, and current type of mental health care) were collected using a self-report questionnaire.

The intensity of psychopathological symptoms was measured by means of the standard version of the Brief Psychiatric Rating Scale (BPRS; Overall and Gorham 1988). This instrument consists of 18 items rated by a clinician on a scale from 1 (symptom not present) to 7 (symptom extremely severe). Summing up individual items generates a total scale score, which can range from 18 to 126, with higher scores denoting more severe symptoms. In our study, the values of Cronbach’s alpha coefficients for the BPRS were .91 in the schizophrenic group, .82 in the affective group, and .91 in the whole sample.

Various aspects of the personal stigma of mental illness were evaluated with the use of the Inventory of Stigmatizing Experiences—Consumer Version (ISE; Stuart et al. 2008). This self-report instrument includes two separate scales—the Stigma Impact Scale (SIS) and the Stigma Experiences Scale (SES). The SIS assesses the severity of the psychosocial impact of stigma—our main variable of interest. This subscale is made up of seven items, of which four ask a respondent how much stigma has affected him/her personally and three ask to what extent stigma has affected a respondent’s family as a whole. Items are scored on an 11-point scale from 0 (reflecting no impact) to 10 (reflecting the highest amount of impact). The sum of all items renders a total score ranging from 0 to 70, with higher scores corresponding to greater impact of stigma. In this study, Cronbach’s alpha coefficient for the SIS was .91 in the schizophrenia group, .96 in the affective group, and .94 in the total sample. According to the authors of the ISE, the other subscale, the SES, evaluates the frequency of stigma experienced. However, a closer analysis of its content reveals that it is rather a complex measure covering several distinct dimensions of personal stigma (Brohan et al. 2010): (1) two items refer to perceived stigma—one of them addresses stereotype awareness, while the other addresses personal fear of encountering stigma; (2) two items concern experienced stigma – one asks about having been teased, bullied or harassed and the other about having been treated unfairly or denied rights; (3) one item regards social withdrawal—a behavioral aspect of self-stigma; (4) five items ask about the impact of stigma on various life domains. Two items assessing perceived stigma are scored on a 5-point Likert-type scale using the response categories of “never”, “rarely”, “sometimes”, “often” and “always”. The response categories for the remaining eight items are “no”, “unsure” and “yes”. All ten items included in the SES are recoded into binary variables: 0 = the absence of stigma (either “never”, “rarely” and “sometimes” or “no” and “unsure”) and 1 = the presence of stigma (either “often” and “always” or “yes”). Items are summed up for a total score ranging from 0 to 10, with higher ratings indicating more stigma. Since, in the current study, we were interested how specific components of personal stigma contribute to stigma impact, we selected for our analyses individual items assessing perceived, experienced and self-stigma and did not use the total score of the SES. Five items of the SES concerning the impact of stigma have been excluded, because their content overlaps to a significant degree with the content of the SIS.

### Procedures

The study was approved by the Bioethical Committee at the IPiN. In each participating service, eligible individuals were identified by staff psychiatrists. They were then approached by the members of the research team, who invited them to take part in the study. All participants provided their informed consent.

### Statistical Analyses

The analyses were carried out with the aid of IBM SPSS Statistics version 21 (SPSS Inc., Chicago, IL). For all study variables, means and standard deviations or percentages, as appropriate, were calculated. Cronbach’s alpha coefficients were computed to ascertain the internal consistency reliability of the instruments. Independent sample *t* tests and Chi square tests were performed for comparing diagnostic groups on continuous and categorical variables, respectively. Hierarchical regression models were used in order to identify factors independently predicting stigma impact as measured by the total score of the SIS. In these analyses, socio-demographic and clinical characteristics were entered as the first set of predictors (Model 1), followed by stigma-related variables, i.e. dichotomized items of the SES assessing perceived, experienced and self-stigma (Model 2). Separate regression analyses were conducted for the whole sample and for both diagnostic groups. Multicollinearity was diagnosed by examining the Variance Inflation Factor (VIF). The VIF values above ten were assumed as indicative of collinearity between the independent variables in the model (Fahrmeir et al. 2013). In all analyses, *P*-values of less than .05 were considered to be statistically significant.

## Results

Socio-demographic and clinical characteristics of schizophrenic and affective groups are presented and compared in Table 1.

**Table 1:** Socio-demographic and clinical characteristics of the participants

Characteristic	Total sample *n* = 229	Schizophrenia *n* = 123	Affective disorders *n* = 106	Group comparison	
				Statistic	*P*-value
Age in years, mean (SD)	45.90 (15.13)	38.92 (12.87)	54.01 (13.46)	*t* = –8.66	<0.001
Sex					
Female, *n* (%)	142 (62.0)	68 (55.3)	74 (69.8)	*χ* ^2^ = 5.10	0.024
Male, *n* (%)	87 (38.0)	55 (44.7)	32 (30.2)		
Education					
Primary or vocational, *n* (%)	37 (16.2)	23 (18.7)	14 (13.2)	*χ* ^2^ = 5.42	ns
Secondary, *n* (%)	94 (41.0)	56 (45.5)	38 (35.8)		
Higher, *n* (%)	98 (42.8)	44 (35.8)	54 (50.9)		
Marital status					
Married^a^, *n* (%)	76 (33.2)	24 (19.5)	52 (49.1)	*χ* ^2^ = 22.41	<0.001
Non-married^b^, *n* (%)	153 (66.8)	99 (80.5)	54 (50.9)		
Living situation					
Living with someone, *n* (%)	173 (75.5)	93 (75.6)	80 (75.5)	*χ* ^2^ = 0.001	ns
Living alone, *n* (%)	56 (24.5)	30 (24.4)	26 (24.5)		
Employment status					
Employed, *n* (%)	89 (38.9)	44 (35.8)	45 (42.5)	*χ* ^2^ = 1.07	ns
Not employed, *n* (%)	140 (61.1)	79 (64.2)	61 (57.5)		
Illness duration in years, mean (SD)	15.36 (12.00)	14.35 (10.71)	16.54 (13.30)	*t* = –1.36	ns
Type of psychiatric setting					
Inpatient ward, *n* (%)	130 (56.8)	84 (68.3)	46 (43.4)	*χ* ^2^ = 14.38	<0.001
Other^c^, *n* (%)	99 (43.2)	39 (31.7)	60 (56.6)		
BPRS total score, mean (SD)	28.50 (11.07)	33.43 (12.20)	22.82 (5.72)	*t* = 8.55	<0.001

*ns* non-significant

^a^ Including by common law; ^b^ Including separated/divorced, widowed and never married; ^c^ Including day ward, outpatient clinic and community mental health center

The schizophrenic group was younger, included a higher percentage of males and a lower percentage of married people. Participants with schizophrenia were also more likely to be hospitalized in inpatient units at the time of assessment and had higher levels of psychopathology as measured by the BPRS. The two diagnostic groups did not differ significantly with respect to education, living situation, employment status and duration of illness.

As shown in Table 2, personal stigma was highly prevalent among respondents. The most common was self-stigma (57 % of the total sample), followed by perceived stigma (47–51 %). Actual stigma experiences were reported by 22–28 % of the participants. In comparison to the affective group, schizophrenia group had significantly higher rates of experienced stigma and self-stigma. No between-group differences were found in the levels of perceived stigma.

**Table 2:** Prevalence of perceived, experienced and self-stigma as measured by selected items of the Stigma Experiences Scale (SES)

	Item	Total sample *n* = 229 % endorsed	Schizophrenia *n* = 123 % endorsed	Affective disorders *n* = 106 % endorsed	Group comparison	
					*χ* ^2^	*P*-value
Perceived stigma	Do you think that people will think less of you if they know you have a mental illness?^a^	47.4	50.8	43.4	1.25	ns
	Do you think that the average person is afraid of someone with a serious mental illness?^b^	51.3	53.3	49.1	0.41	ns
Experienced stigma	Have you ever been teased, bullied, or harassed because you have a mental illness?^c^	27.6	35.2	18.9	7.61	0.006
	Have you felt that you have been treated unfairly or that your rights have been denied because you have a mental illness?^d^	21.9	28.7	14.2	7.00	0.008
Self-stigma	Do you try to avoid situations that may be stigmatizing to you?^e^	56.8	63.4	49.1	4.78	0.029

The percentages of participants endorsing SES items refer to those who responded “often” or “always” to items assessing perceived stigma or “yes” to items assessing experienced stigma and self-stigma

*ns* non-significant

^a^Short: personal fear of stigma; ^b^ short: stereotype awareness; ^c^ short: being teased, bullied or harassed; ^d^ short: being treated unfairly or denied rights; ^e^ short: social withdrawal

The mean score (*M*) on the SIS for the entire sample was 21.21 (standard deviation [*SD*] = 20.75). The independent sample *t* test revealed that people with schizophrenia reported a significantly higher impact of stigma than people with affective disorders (*M* = 28.33, *SD* = 19.36 vs *M* = 12.94, *SD* = 19.27; *t* = 6.01, *P* < .001). The results of the multiple regression analyses predicting stigma impact are displayed in Table 3.

**Table 3:** Summary of hierarchical multiple regression analyses predicting stigma impact in the whole sample and in the diagnostic subgroups

Predictor	Total sample *n* = 229		Schizophrenia *n* = 123		Affective disorders *n* = 106	
	Model 1	Model 2	Model 1	Model 2	Model 1	Model 2
Gender^a^	−0.08	−0.02	−0.13	−0.04	−0.09	−0.03
Age	−0.12	−0.06	0.04	0.01	−0.26*	−0.07
Education^b^						
Primary or vocational	0.06	0.07	0.15	0.14	−0.01	−0.03
Higher	−0.06	−0.06	−0.19	−0.15	−0.01	−0.08
Marital status^c^	−0.01	0.01	0.13	0.16	−0.20	−0.10
Living situation^d^	0.04	0.06	0.10	0.11	−0.05	0.04
Employment status^e^	−0.04	−0.01	−0.03	−0.01	−0.04	0.02
Duration of illness	0.12	0.06	0.15	0.08	0.04	0.04
Type of psychiatric setting^f^	−0.21**	−0.16*	−0.13	−0.09	−0.30*	−0.27*
BPRS total score	0.04	0.08	−0.01	0.02	0.01	0.12
Diagnosis^g^	−0.25**	−0.16*	–	–	–	–
Stigma-related variables						
Personal fear of stigma	–	0.08	–	−0.01	–	0.08
Stereotype awareness	–	0.11	–	0.18	–	0.11
Being teased, bullied or harassed	–	0.15*	–	0.15	–	0.13
Being treated unfairly or denied rights	–	0.20**	–	0.13	–	0.42***
Social withdrawal	–	0.18**	–	0.20*	–	0.16
*R* ^*2*^	0.227	0.394	0.142	0.252	0.242	0.535
Δ*R* ^*2*^	–	0.167	–	0.110	–	0.293

Standardized regression coefficients (Beta) are presented

*BPRS* brief psychiatric rating scale

^a^ 0 = Female, 1 = male

^b^ Secondary = reference category

^c^ 0 = Non-married (including separated/divorced, widowed and never married), 1 = married (including by common law)

^d^ 0 = Living with someone, 1 = living alone

^e^ 0 = Not employed, 1 = employed

^f^ 0 = Inpatient ward, 1 = other (including day ward, outpatient clinic and community mental health center)

^g^ 0 = Schizophrenia, 1 = affective disorders

* *P* < .05; ** *P* < .01; *** *P* < .001

In the whole sample, the factors significantly associated with a greater impact of stigma were schizophrenia diagnosis, current inpatient treatment, experienced stigma (being teased, bullied or harassed and being treated unfairly or denied rights), and self-stigma (social withdrawal). A total of 39.4 % of the variance in stigma impact was explained (22.7 % by socio-demographic and clinical variables entered in the first step and an additional 16.7 % by stigma-related variables entered in the subsequent step). For the schizophrenia group, only self-stigma (social withdrawal) significantly contributed to a stronger stigma impact. In the affective group, a more severe impact of stigma was significantly predicted by inpatient status and experienced stigma (being treated unfairly or denied rights). Older age predicted lesser stigma impact among participants with mood disorders in Model 1, but this association was no longer significant when stigma-related variables were introduced into the regression equation. While as much as 53.5 % of the variance in the dependent variable was explained in the subgroup with affective disorders (24.2 % by socio-demographic and clinical variables and 29.3 % by stigma-related variables), in the subsample with schizophrenia only 25.2 % of the variance was accounted for (14.2 % by socio-demographic and clinical variables and 11 % by stigma-related variables). The VIF values ranged from 1.09 to 3.17, indicating no collinearity problems in the models.

## Discussion

This study found, as expected, that people with schizophrenia reported greater impact of stigma on personal and family life than people with affective disorders. Importantly, this was true even after controlling for socio-demographic and clinical background characteristics, severity of psychopathology as well as perceived, experienced and self-stigma. Therefore, the observed difference in the intensity of stigma impact cannot be totally accounted for simply by the fact that participants with schizophrenia were more psychiatrically impaired or that they internalized stigma and experienced social rejection more frequently than participants with affective disorders. Rather, it seems reasonable to hypothesize that persons with mood disorders may be more resistant to the detrimental effects of stigma. This supposition is supported by the results of the study by Sarisoy et al. (2013), who found that individuals with bipolar affective disorder had higher levels of stigma resistance in comparison to those with schizophrenia. It is recommended that future research should explore in more detail whether people with various mental illnesses differentially react to stigma and, if this is the case, how it affects the psychosocial consequences of stigma.

Apart from diagnosis, the only clinical variable significantly associated with stigma impact in the whole sample was current type of mental health care. Namely, participants from inpatient wards reported a stronger impact of stigma than those under outpatient, community or day care. Although previous studies yielded inconclusive results regarding the relationships of personal stigma and type of psychiatric setting (Gerlinger et al. 2013; Livingston and Boyd 2010), our finding is consistent with a general conviction that psychiatric inpatient hospitalization is a particularly devastating stigmatization experience (Falk 2001). Interestingly, when analyzed in the two diagnostic categories separately, the inpatient status proved to contribute significantly to the impact of stigma in the affective disorders group, but not in the schizophrenia group. This may be due to the fact that inpatient wards, in which the data for this study was collected, are designated primarily to provide care for people with acute exacerbations and relapses of psychotic illnesses. It is possible, given this, that entering such facilities can increase the sense of differentness and isolation of persons with mood disorders. Furthermore, the treatment programs employed in these settings may better address the needs of persons with psychosis than those of persons with affective disorders. It is yet to be investigated whether this is an inpatient hospitalization as such or rather specific characteristics of inpatient treatment settings that add to the burden of stigma among people diagnosed with depressive or bipolar disorders.

As far as stigma-related variables are concerned, experienced stigma and self-stigma, but not perceived stigma turned out to be significant predictors of stigma impact in the entire sample. This pattern of results is not very surprising, since perceived stigma is regarded as the initial stage of the process of self-stigmatization, which becomes harmful only when public stereotypes about people with mental illness are accepted and internalized by the individual (Corrigan et al. 2006). It is more notable that the two diagnostic groups included in our study differed with respect to which dimensions of personal stigma contributed most to the impact of stigma. Among respondents with schizophrenia stigma impact was significantly predicted solely by self-stigma, whereas among the affective group it was experienced stigma that was identified as a crucial determinant of psychosocial consequences of stigma. This difference merits further investigation since it may point to the distinct patterns of personal vulnerability/resilience factors influencing the effectiveness of dealing with various components of personal stigma in either diagnostic group. Identifying these factors may reveal relevant targets for programs aimed at reducing the psychosocial harms caused by stigma among persons with schizophrenia and mood disorders.

Unexpectedly, the intensity of psychopathological symptoms and illness duration turned out to be unrelated to stigma impact. This indicates that the psychosocial impact of stigma is not a direct consequence of psychiatric impairment or length of exposure to stigmatizing events. Our results also confirm the conclusions from two recent literature reviews (Gerlinger et al. 2013; Livingston and Boyd 2010) that socio-demographic variables are not strongly correlated with the personal stigma of mental illness.

The present study may have relevant practical implications. The findings add to the extant literature demonstrating that self-stigma and experienced stigma can negatively influence personal and family life. Hence, counteracting their pernicious effects is crucial for the improvement of the well-being of people with mental health problems. On the basis of a review of empirical studies of self-stigma reduction strategies, two prominent approaches in this area can be recommended: interventions directed at correcting the stigmatizing beliefs and attitudes of the individual, and interventions attempting to enhance the individual’s skills for coping with self-stigma through improvements in self-esteem, empowerment, and help-seeking behavior (Mittal et al. 2012). Anti-stigma efforts should also include interventions aiming to help people with mental illnesses to work out useful strategies for dealing with social rejection and a devalued social identity. From a recent longitudinal study by Ilic et al. (2014), which tested the effectiveness of a wide array of stigma-coping strategies, two of them, i.e. selective disclosure and information seeking, emerged as the most beneficial: the former predicted less stigma experiences and both predicted better mental health at follow-up. In view of these findings, clinicians should assist people with mental health problems in their decisions about the manner and extent of disclosure and support them in their efforts to gain better knowledge and understanding of their illness. If other studies consistently replicate our finding that it is self-stigma that is a core predictor of stigma impact in schizophrenia, whereas in mood disorders it is experienced stigma, then this should be reflected in the main focus of the programs targeting personal stigma designed for people with these diagnoses. Our results also point to the importance of the type of psychiatric care in the stigmatization process and support the notion that inpatient treatment may have severe adverse effects. This seems particularly relevant for individuals with mood disorders. Obviously, there are situations where inpatient hospitalization cannot be avoided, but in such cases intensive efforts should be undertaken to minimize its stigmatizing impact. These should include: first, improving the quality of inpatient care and increasing patients’ satisfaction with received services by responding to their needs and preferences, which should be assessed on a regular basis; and, second, incorporating anti-stigma interventions into the therapeutic programs delivered in inpatient facilities.

Several limitations of the current study must be recognized. First of all, the cross-sectional nature of the data precludes making definitive statements about the direction of the observed relationships. Our participants were recruited from just one psychiatric institution, so they may not be representative of the entire population of people with mental illness in Poland. Next, in order to increase the statistical power and reduce the number of analyses needed, we combined depressive episodes, recurrent depressive disorders and bipolar disorders into one broad diagnostic category of affective disorders; in the future, it would be useful to compare the intensity and correlates of the impact of stigma among more homogenous diagnostic groups. Furthermore, our measure of stigma impact has not yet been fully tested psychometrically. In this study, Cronbach’s alpha for the SIS was above .90, which may indicate some redundancy across items (Streiner 2003). It is, then, worth considering whether the instrument can be shortened. The SIS still needs to be validated against alternative measures of the effects that stigma has on persons with mental disorders, such as e.g. the Stigma Stress Scale (Rüsch et al. 2009, 2014), which evaluates perceived stigma-related harm exceeding the perceived coping resources of the individual. Another limitation is that we used individual dichotomized items as proxy measures of dimensions of personal stigma predicting stigma impact. Thus, our findings need to be replicated using more complex, psychometrically robust instruments for the assessment of the specific elements of personal stigma, such as e.g. the Perceived Devaluation-Discrimination Scale (PDD; Link 1987) for assessing perceived stigma, the Discrimination and Stigma Scale (DISC; Brohan et al. 2013) for assessing experienced stigma, or the Internalized Stigma of Mental Illness Scale (ISMI; Ritsher et al. 2003) for measuring self-stigma. It should be noted, as well, that our set of predictors explained twice as much of the variance in stigma impact in the affective group as it did in the schizophrenia group. This difference in the predictive value of our models in the two diagnostic groups raises the possibility that other variables, which were not included in this research, may play an important role in modifying the psychosocial impact of stigma among people with schizophrenia. Some unmeasured factors may also have affected the observed difference in the severity of stigma impact between diagnostic categories. For example, we did not collect data on the number of previous inpatient admissions. It is probable that participants with schizophrenia had experienced more hospitalizations in inpatient settings than those with mood disorders. The same applies to past experiences with compulsory treatment, which has been found to be associated with various aspects of the personal stigma of mental illness (Rüsch et al. 2014; Thornicroft et al. 2009).

Given the above limitations, our analyses should be regarded as preliminary and exploratory. Despite this, the results obtained contribute to a better understanding of the factors influencing the psychosocial consequences of psychiatric stigma and underscore the role of the type of mental illness in the process of stigmatization. Researchers and clinicians elaborating anti-stigma interventions should take into consideration these factors and address the specific features of stigma associated with various psychiatric diagnoses.
